# 

**Published:** 1862-01

**Authors:** 


### CONTENTS.

ORIGINAL ESSAYS AND COMMUNICATIONS.

The Application of Chemistry to Dentistry. By CHARLES M. WETHERILL, PH., D., M.D.. 5

Who are Dentists? By J. ALLEN, D. D. S. 22

The Warping of Plates. By DR. J. H. HYDE, of Lewistown, Ill. 27

CORRESPONDENCE.

The Dental College 28
The Baltimore College 28
Dental Periodicals 28
S. Driggs 31

PROCEEDINGS OF SOCIETIES..

Michigan Dental Association 32

SELECTIONS.

Water: Its History, Characteristics, Hygienic, and Therapeutic Uses. By S. W. FRANCIS, A. M. M. D., 37
India-Rubber Stoppers for Bottles 42
A New Caustic for Toothache 43

EDITORIAL.

